# Neurocysticercosis: An Easy to Miss Diagnosis in Non-Endemic Regions

**DOI:** 10.7759/cureus.12066

**Published:** 2020-12-13

**Authors:** Akshit Chitkara, Sarah Chhabra, Akshit Griwan, Naman Khurana, Piyush Puri

**Affiliations:** 1 Internal Medicine, Sh. Moolchand Super Speciality Hospital, Karnal, IND; 2 Internal Medicine, Mount Sinai Medical Center, Miami, USA; 3 Internal Medicine, East Tennessee State University Quillen College of Medicine, Mountain Home, USA; 4 Obstetrics and Gynecology, Sanjay Gandhi Hospital, Delhi, IND; 5 Internal Medicine, Al Flah School of Medical Sciences and Research Center, Faridabad, IND

**Keywords:** neuro-surgery, neuro-imaging

## Abstract

A 29-year-old male presented with swollen gums and stomatitis for the past two months. History revealed that he had moved to the United States from India six years ago and had a first episode of generalized tonic-clonic seizure with confusion and loss of consciousness. Meningioma of the brain was diagnosed, and a Gamma Knife excision of the meningioma was planned. The patient refused to proceed with the surgery and came back to India for a second opinion. Upon repeat MRI scan, the neurosurgeon revised the diagnosis to neurocysticercosis (NCC), and the patient was treated with albendazole, prednisolone, and phenytoin and recovered completely. Hence an unnecessary brain surgery was avoided. The complaint of stomatitis and gingival hypertrophy was due to the side effects of phenytoin.

NCC remains a major public health problem in developing countries, and it should be considered as a differential diagnosis in patients from NCC endemic regions.

## Introduction

Neurocysticercosis (NCC) is the most severe form of cysticercosis, an infection caused by ingesting tapeworm (Taenia solium) eggs. The larvae may travel to the brain via the bloodstream and develop into NCC. It is a very common disorder in the Indian subcontinent, Latin America, Africa, and China (1:1000) and is responsible for about 30% of total cases of epilepsy [1-3]. In developed countries, it is a comparatively rare disorder, so it is more often misdiagnosed or missed while making the differential diagnosis [4].

The radiological (MRI/contrast-enhanced CT) features of NCC are quite similar to those of brain abscess, tuberculoma, meningioma, brain metastasis, or hydatid cyst, leading to wrong interpretation of the brain lesion [5]. Hence the geographic background and travel history of the patient must be evaluated while analyzing the lab work-up and neuroimaging data.

## Case presentation

A 29-year-old male presented with swollen gums and stomatitis for the past two months. The patient denied throat pain, fever, upper respiratory tract infection (URTI), diarrhea, dysentery, abdominal pain, or any urinary problem. There was no history of diabetes mellitus, hypertension, or coronary artery disease. He was a non-smoker and non-alcoholic. There was no history of recreational drug use or tobacco chewing. He consumed mildly spicy food and around two cups of tea or coffee per day. He was obese with a BMI of 28.

**Figure 1:**
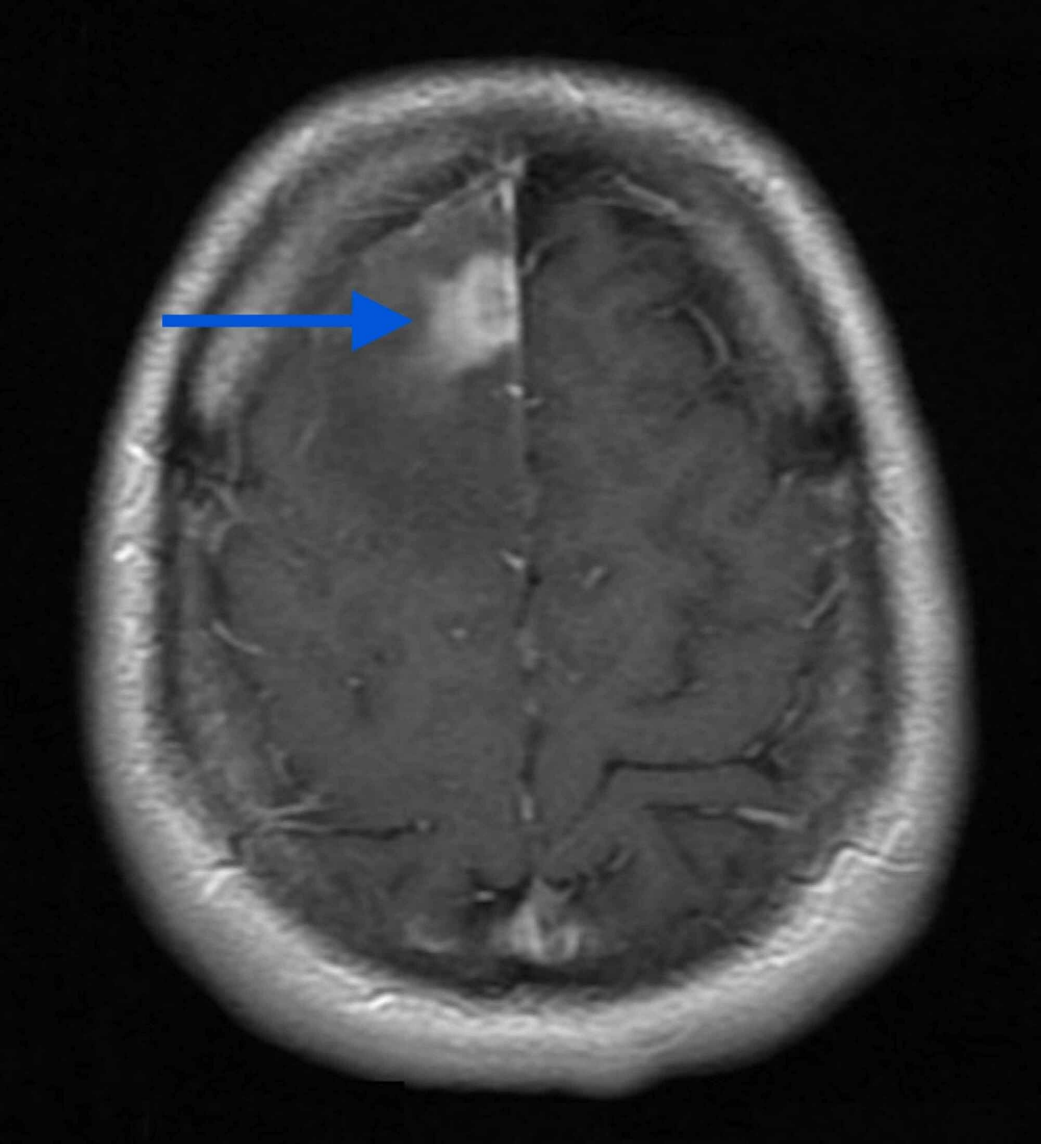
Axial post-contrast T1 MRI showing 16×14×21 mm conglomerate ring-enhancing lesion in the paramedian region of the right frontal lobe with surrounding edema.

**Figure 2:**
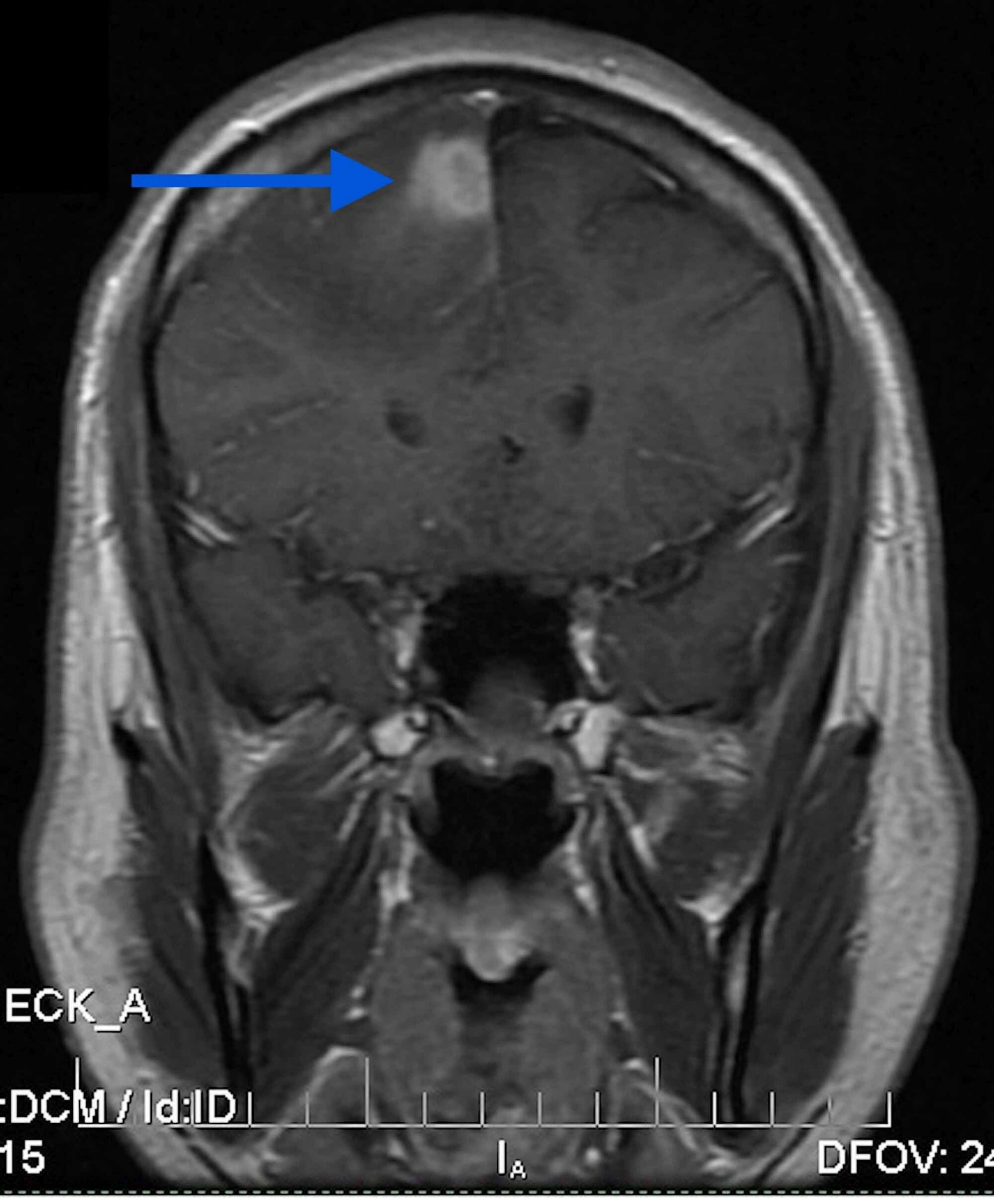
Coronal post-contrast T1 MRI showing 16×14×21 mm conglomerate ring-enhancing lesion in the paramedian region of the right frontal lobe with surrounding edema.

**Figure 3:**
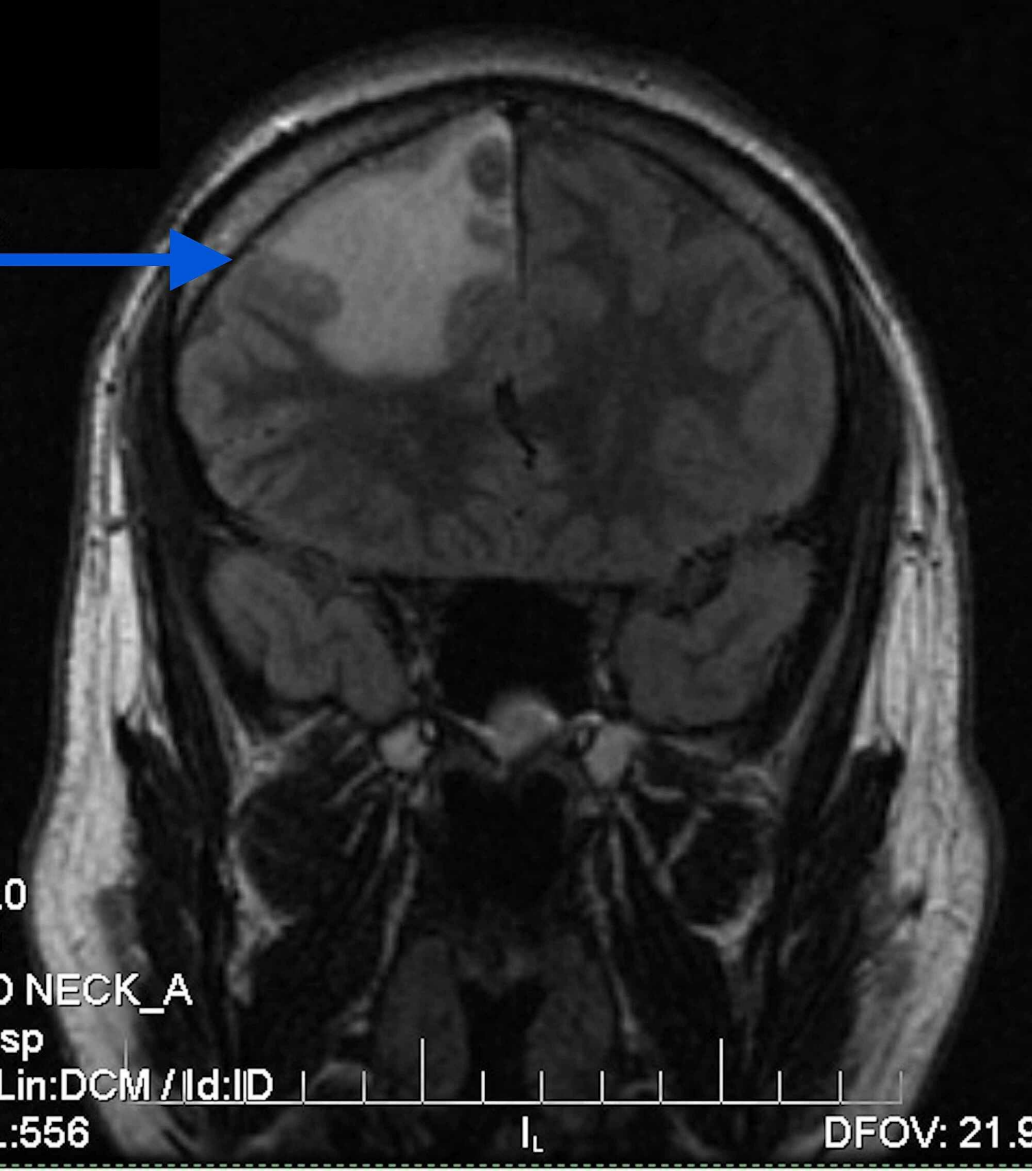
Coronal T2 FLAIR MRI showing 16×14×21 mm conglomerate ring-enhancing lesion in the paramedian region of the right frontal lobe with surrounding edema. FLAIR: fluid-attenuated inversion recovery

On further interrogation, the patient revealed that six years ago, when he was 23 years old, he had moved to the US, from India, for higher education. Eight months after his move, he suffered a generalized tonic-clonic seizure with loss of consciousness that lasted for 10 minutes. Upon a 911 call, he was immediately hospitalized, and fosphenytoin and prednisolone were started. After regaining consciousness, the patient had confusion but no neurological deficit. After laboratory workup and neuroimaging, a final diagnosis of meningioma of the right frontal lobe of the brain was established, and surgical resection was planned (Figures 1-3).

**Figure 4:**
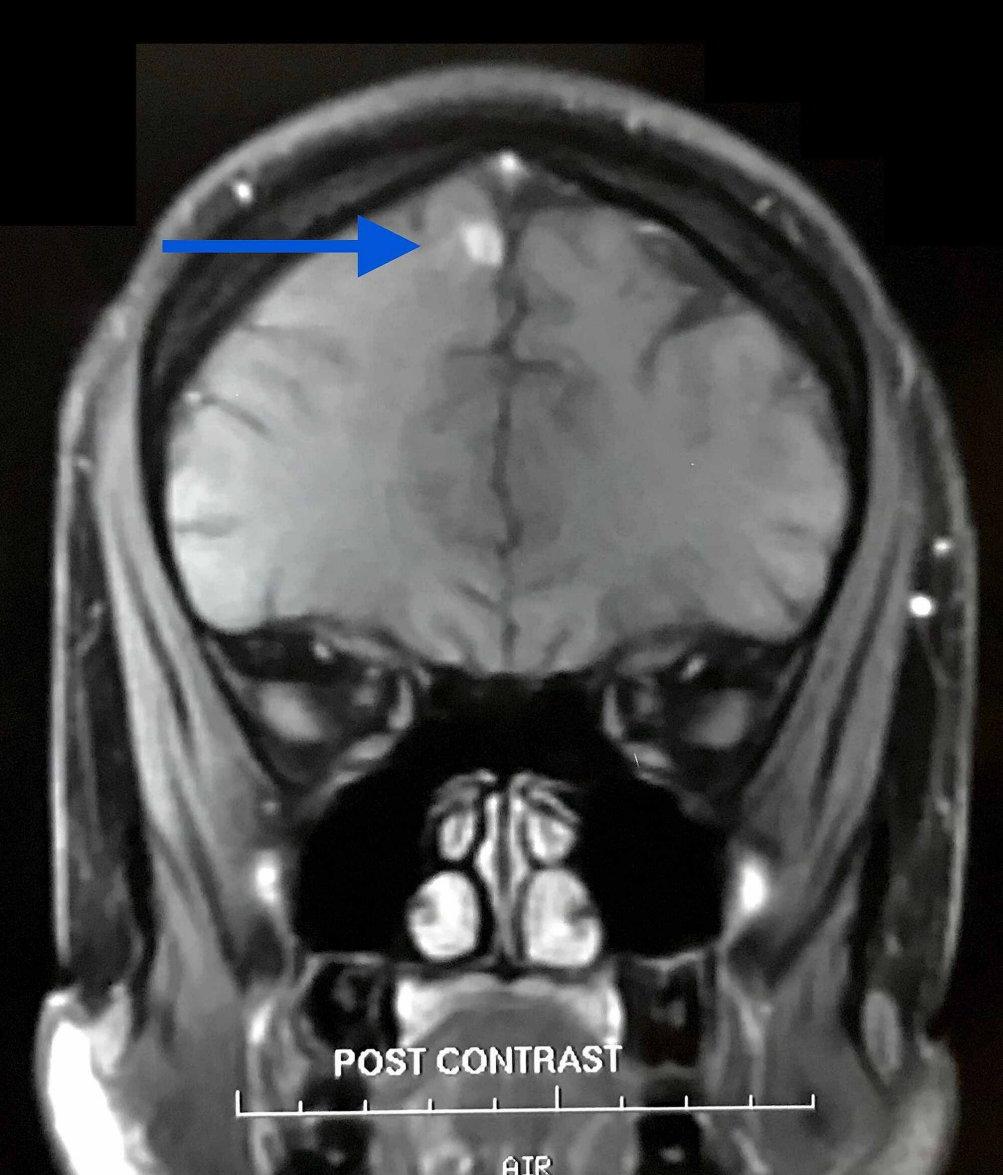
Neurocysticercosis in the right frontal lobe of the brain, post-contrast coronal MRI.

**Figure 5:**
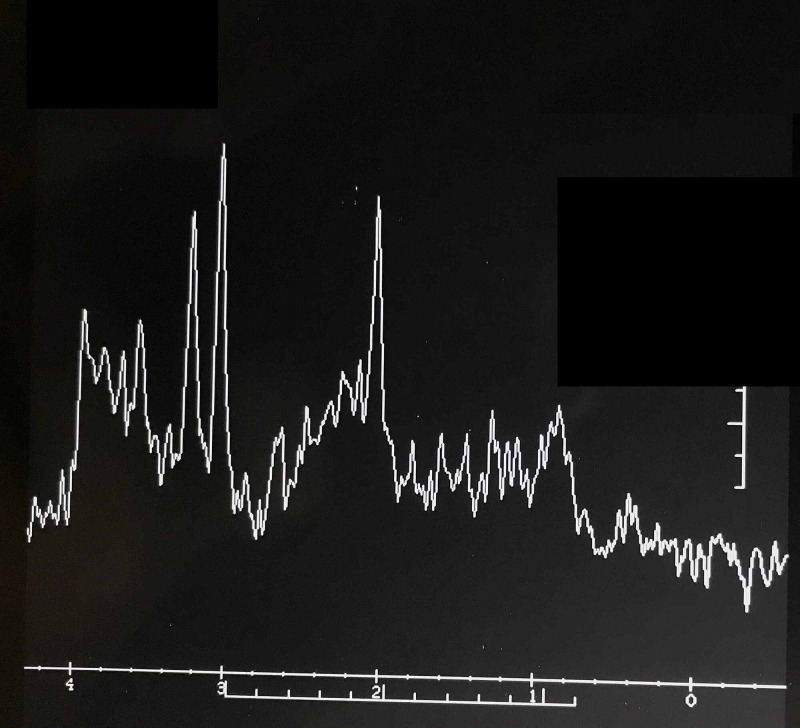
MR spectroscopy through the lesion shows normal Creatine (Cr), Choline (cho), and N-acetylaspartate (Naa) ratio with no abnormal increase in Myo-inositol (MI) or any lipid or lactate peak.

**Figure 6:**
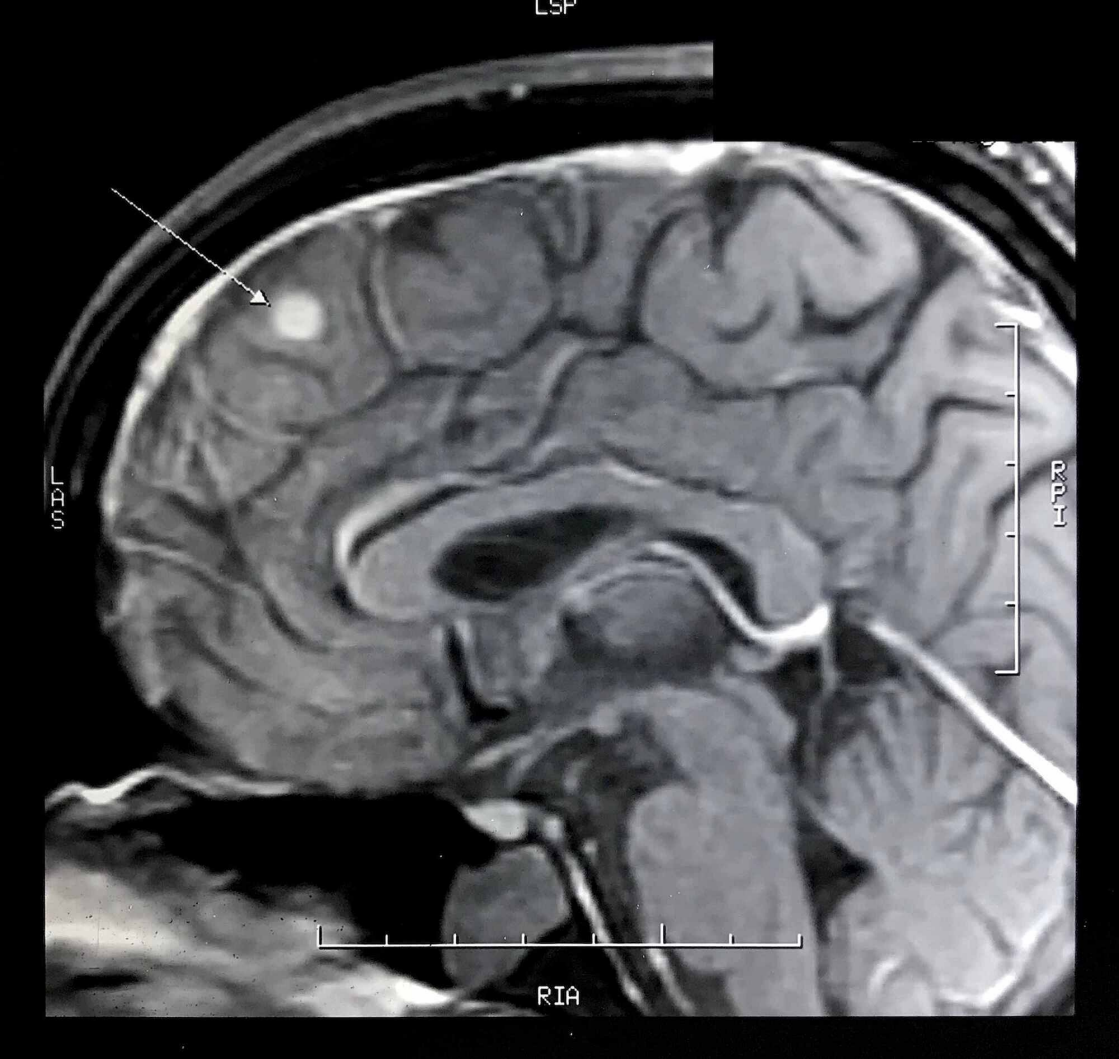
Neurocysticercosis in the right frontal lobe of the brain on MRI.

The patient refused to proceed with the surgery and came back to India after two weeks for a second opinion. The neurosurgeon in India reordered neuroimaging. To confirm the diagnosis, two MRIs were conducted by two separate radiologists. They compared the older scan with the new MRI studies and revised the diagnosis to NCC (Figures 4-6).

Investigations

MRI study of the US hospital showed a 16×14×21 mm conglomerate ring-enhancing lesion in the paramedian region of the right frontal lobe with surrounding edema (Figures 1-3).

MRI study done in India revealed nodular intraparenchymal disc enhancing lesions in the paramedian region of the right frontal lobe, 7×7×6 mm in size. It was hypointense on T2 weighted image (T2WI), and a moderate degree of edema was noted around the lesion. There was no midline shift. The final impression confirmed the presence of a degenerating NCC in the right frontal lobe of the brain (Figures 4, 6).

Laboratory results for the complaints of gingivitis were as follows: Haemoglobin 14.4 g/dL, white blood cells 5,700 per microliter, platelet count 242,000 per microliter, neutrophil 57%, basophil 0.4%, lymphocyte 36.6%, monocyte 5.3%, eosinophil 0.7%, blood glucose 122 mg/dL, blood urea nitrogen (BUN) 10 mg/dL, serum creatinine 1.02 mg/dL, serum calcium 9.6 mg/dL, alkaline phosphatase 64 U/L, serum phenytoin level <1.0 mcg/ml.

Differential diagnosis

Establishing the diagnosis of NCC is not always a straightforward process. NCC, brain abscess, tuberculoma, hydatid cyst, malignancy, fungal infections, syphilis, sarcoidosis, or brain metastasis may look alike on neuroimaging in the form of small nodular intraparenchymal disc enhancing lesion [5]. Hence the diagnosis may be difficult even with neuroimaging and serological studies.

NCC is the most common parasitic disease of the central nervous system in the Indian subcontinent along with Latin America, Africa, and China [2,3]. Therefore geographic background and travel history of the patient must be taken into account before finalizing the diagnosis for such patients.

Outcome and follow-up

There are multiple side effects of phenytoin therapy [6,7]. The patient has been asymptomatic and seizure-free since the initial occurrence. 

## Discussion

Approximately 25% of cases of epilepsy in India are due to NCC [8]. In 2011, it was estimated that NCC associated active epilepsy led to an annual median loss of US $185.14 million, leading to significant health and economic impact in India [9]. The pork tapeworm (Taenia solium) infects the human nervous system and skeletal muscles. These larvae enter the bloodstream and settle down in the brain, retina, subcutaneous tissue, or skeletal muscle. The larvae evoke a cellular reaction starting with infiltration of neutrophils, eosinophils, lymphocytes, plasma cells, and at times giant cells. It produces a granuloma and may remain alive for multiple years [10]. They may die, and the lesion may get calcified. In addition to epilepsy, the granuloma may lead to a focal neurological deficit, visual disturbances, behavioral disorders, or obstructive hydrocephalus.

This disease is one of the main causes of epileptic seizures in many less developed countries. It is also increasingly seen in more developed countries because of immigration from endemic areas. NCC is extremely prevalent in Latin America, the Indian subcontinent, Sub-Saharan Africa, coastal North Africa, and China [2,3]. Open field defecation, dirty toilets, poor sanitation are the main culprits for the excessive prevalence of this disease [11].

The lesions in the brain on neuroimaging may resemble those of hydatid cyst, tuberculoma, meningioma, brain abscess, or brain metastasis. Eosinophilia in blood or cerebrospinal fluid (CSF) and enzyme-linked immunosorbent assay (ELISA) testing may help, but neuroimaging is the mainstay for diagnosing NCC [12].

NCC usually does not require any surgery. Albendazole 15 mg per kg per day or praziquantel 50 mg per kg per day for two to four weeks are very good larvicidal drugs [13]. Prednisolone 30 mg per day for two to four weeks helps in reducing the inflammatory edema around the lesion and may be tapered gradually over the next two to three weeks [14]. Antihelminthic treatment, together with anticonvulsants, can reduce seizure activity by greater than 90%, and anticonvulsants should be given for a minimum of three years, extendable to five years or lifetime, subject to recovery and seizure-free period [15].

## Conclusions

Detailed history taking is the most important part of any patient's workup. Personal history, family history, history of travel, and immigration are very important in today's culturally diverse and fast-moving world, and more so when infective pathology is a part of the differential diagnosis. By being more vigilant and thorough, we can safeguard the patients from unnecessary surgical procedures and their sequelae. Side effects such as gum hypertrophy should not deter a physician from using phenytoin in the treatment of epilepsy. There should be proper monitoring of the phenytoin levels to avoid its toxicity as it has a narrow therapeutic index and wide dose-related side effect profile.
